# Interference with TGFβ1-Mediated Inflammation and Fibrosis Underlies Reno-Protective Effects of the CB1 Receptor Neutral Antagonists AM6545 and AM4113 in a Rat Model of Metabolic Syndrome

**DOI:** 10.3390/molecules26040866

**Published:** 2021-02-06

**Authors:** Basma G. Eid, Thikryat Neamatallah, Abeer Hanafy, Hany M. El-Bassossy, Lenah Binmahfouz, Hibah M. Aldawsari, Atif Hasan, Gamal Abd El-Aziz, Kiran Vemuri, Alexandros Makriyannis

**Affiliations:** 1 Department of Pharmacology and Toxicology, Faculty of Pharmacy, King Abdulaziz University, Jeddah 21589, Saudi Arabia; Taneamatallah@kau.edu.sa (T.N.); ahanafyvet@yahoo.com (A.H.); lbinmahfouz@kau.edu.sa (L.B.); 2 Department of Pharmacology, Faculty of Veterinary Medicine, Kafrelsheikh University, Kafrelsheikh 33516, Egypt; 3 Department of Pharmacology and Toxicology, Faculty of Pharmacy, Zagazig University, Zagazig 44519, Egypt; helbassossy@pharmacy.zu.edu.eg; 4 Department of Pharmaceutics, Faculty of Pharmacy, King Abdulaziz University, Jeddah 21589, Saudi Arabia; haldosari@kau.edu.sa; 5 Department of Anatomy and Embryology, Faculty of Veterinary Medicine, Kafrelsheikh University, Kafrelsheikh 33516, Egypt; atifhasan178@gmail.com; 6 Department of Anatomy, Faculty of Medicine, King Abdulaziz University, Jeddah 21589, Saudi Arabia; dr_gamal_said@yahoo.com; 7 Center for Drug Discovery, Northeastern University, Boston, MA 02115, USA; kiranvvemuri@gmail.com (K.V.); a.makriyannis@northeastern.edu (A.M.); 8 Departments of Chemistry and Chemical Biology and Pharmaceutical Sciences, Northeastern University, Boston, MA 02115, USA

**Keywords:** metabolic syndrome, cannabinoids, AM6545, AM4113, kidney, rats

## Abstract

The role of cannabinoid receptors in nephropathy is gaining much attention. This study investigated the effects of two neutral CB1 receptor antagonists, AM6545 and AM4113, on nephropathy associated with metabolic syndrome (MetS). MetS was induced in rats by high-fructose high-salt feeding for 12 weeks. AM6545, the peripheral silent antagonist and AM4113, the central neutral antagonist were administered in the last 4 weeks. At the end of study, blood and urine samples were collected for biochemical analyses while the kidneys were excised for histopathological investigation and transforming growth factor beta 1 (TGFβ1) measurement. MetS was associated with deteriorated kidney function as indicated by the elevated proteinuria and albumin excretion rate. Both compounds equally inhibited the elevated proteinuria and albumin excretion rate while having no effect on creatinine clearance and blood pressure. In addition, AM6545 and AM4113 alleviated the observed swelling and inflammatory cells infiltration in different kidney structures. Moreover, AM6545 and AM4113 alleviated the observed histopathological alterations in kidney structure of MetS rats. MetS was associated with a ten-fold increase in urine uric acid while both compounds blocked this increase. Furthermore, AM6545 and AM4113 completely prevented the collagen deposition and the elevated expression of the TGFβ1 seen in MetS animals. In conclusion, AM6545 and AM4113, possess reno-protective effects by interfering with TGFβ1-mediated renal inflammation and fibrosis, via peripheral action.

## 1. Introduction

Metabolic syndrome (MetS), is a combination of metabolic abnormalities that commonly manifests as insulin resistance (IR), abdominal obesity, dyslipidemia and high blood pressure [1]. Patients with MetS are at a high risk of developing diabetes, atherosclerotic cardiovascular disease (CVD) and renal impairment [2]. All of these diseases represent serious and often fatal health conditions that are prevalent in most countries. The prevalence of MetS is increasing globally with over a billion people now affected [3]. This increase is associated with physical inactivity, high-calorie food consumption and drinks supplemented with sugars [4,5]. The relationship between MetS and chronic kidney disease (CKD) is controversial. However, several studies have reported that a fructose-rich diet induces MetS that subsequently progresses into various manifestations of nephropathy such as reduced glomerular filtration, albuminuria, uricosuria, proteinuria and changes in renal morphology [6,7,8]. Possible mechanisms of renal impairment may include the systemic release of pro-inflammatory cytokine mediators, free radicals and oxidative stress in MetS [9,10]. Indeed, the incidence of CKD in patients with MetS is 2.6-fold higher than individuals without any MetS components [11].

Therapeutic interventions for MetS include modulation of the overactive endocannabinoid (EC) system through inhibition of the cannabinoid CB1 receptor subtype [12]. Inhibition of CB1 receptor has been demonstrated to regulate appetite, food intake and lipogenesis in obese rats [13,14,15]. The CB1 is a class A Rhodopsin-like G-protein coupled receptor (GPCR) that activates mainly Gαi/o proteins [16]. The ubiquitous CB1 receptors are vastly found in the central nervous system (CNS), which may increase the incidence of neuropsychiatric disorders [17]. It has been established that a full system of endocannabinoids exists in the kidney and has a significant role in renal homeostasis as well as in the development of diabetic nephropathy and conditions such as CKD [18]. CB1 and CB2 receptors were reported to be present in the various parts of the nephron as well as other renal cells in both humans and rodents [19]. Furthermore, the endocannabinoid system in the kidney plays a predominant role in renal hemodynamics [20].

Rimonabant (SR141716), for example, is a CB1 receptor inverse agonist that was approved clinically in April 2006 to treat metabolic complications and obesity in Europe [21]. However, withdrawal of the CNS penetrating drug rimonabant was pursued by the European Medicines Agency since it was associated with anxiety, depression and increased tendency to cause suicide [17].

To circumvent the neuropsychiatric side effects of rimonabant, which were attributable to the central effects associated with its inverse agonist profile, we pursued two strategies for the development of efficacious analogs that lack its undesirable side effects. The first of these was the discovery of CB1 neutral antagonists. These compounds, unlike their inverse agonist counterparts that act by promoting the inactive state of the receptor, prevent the endogenously occurring endocannabinoids from interacting with CB1. AM4113 represents a successful CB1 neutral antagonist that shares the desirable effects of rimonabant but lacks it undesirable side effects. Our second strategy was to develop CB1 receptor antagonists that are unable to cross the blood brain barrier. AM6545 represents a successful peripherally acting ligand, while at the same time maintaining the CB1 neutral antagonist profile. Both drugs were reported to retain the therapeutic activity in different animal models with less adverse effects than rimonabant [22,23,24,25,26].

In the current study, we aimed to determine whether treatment with the CB1 receptor antagonists AM6545 and AM4113 would improve kidney function in a high-fructose high-salt model of metabolic syndrome, and to explore the underlying mechanism for the improved function. Such a study would allow us to identify if any of the effects of these compounds have a component related to centrally produced CB1 inactivation.

## 2. Results

### 2.1. Effect of AM6545 and AM4113 Treatments on Renal Endocannabinoid Tone

Measurement of anandamide (AEA) and 2-arachidonoylglycerol (2-AG) in kidney homogenates was performed in order to assess the endocannabinoid tone in the kidney. As shown in Table 1, when control rats (C) were treated with either AM6545 (C + A65) or AM4113 (C + A41) there was no significant effect on the content of both AEA and 2-AG in the kidney. Animals with metabolic syndrome (M) displayed elevated levels of both AEA and 2-AG compared to controls (C). After treating the metabolic syndrome rats with either AM6545 (M + A65) or AM4113 (M + A41), the animals still showed much higher levels of both AEA and 2-AG in the kidneys relative to the controls.

### 2.2. Effect of AM6545 and AM4113 Treatments on Renal Function in MetS Rats

**Figure 1:**
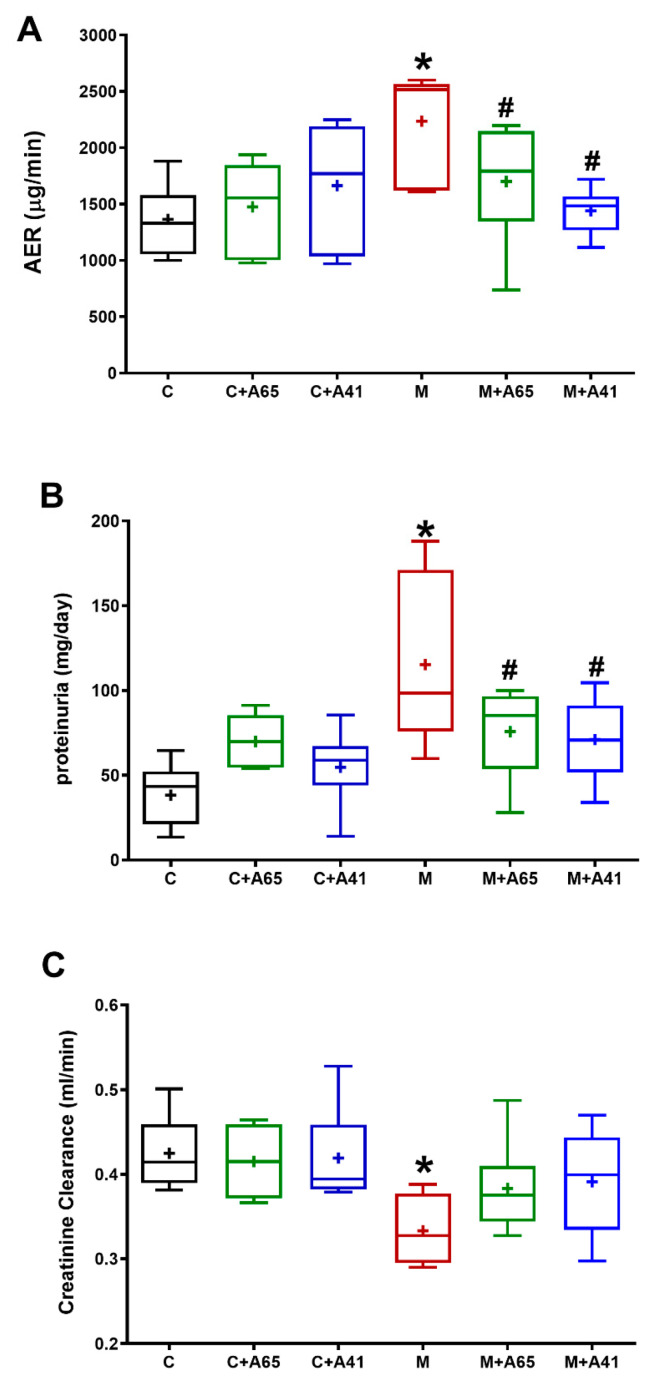
The CB1 receptor antagonists AM6545 and AM4113 improve renal functional parameters in high-fructose high-salt fed metabolic syndrome rats. (**A**) shows the albumin excretion rate (AER), (**B**) shows the proteinuria and (**C**) shows the creatinine clearance measured in control (C) and metabolic syndrome (M) rats as well as after 4 weeks of intraperitoneal injection of AM6545 in control (C + A65) and metabolic rats (M + A65) or AM4113 in control (C + A41) or metabolic rats (M + A41) at a dose of 10 mg/kg/day. Results are shown as box plots and the mean is shown as (+) (*n* = 8). * *p* < 0.05 relative to the control group and # *p* < 0.05 relative to the metabolic syndrome group by one-way ANOVA followed by Tukey’s post hoc test.

MetS due to high-fructose high-salt feeding is associated with deterioration of the kidney function as indicated by the increased albumin excretion rate (AER) and proteinuria in comparison to controls (*p* < 0.05, Figure 1A,B). Both AM6545 and AM4113 significantly inhibited (*p* < 0.05) the elevated proteinuria and albumin excretion rate to similar levels suggesting protection of renal function in MetS. Neither AM6545 nor AM4113 significantly affected AER or proteinuria when administered in control animals (Figure 1A,B). Creatinine clearance was calculated and was much lower in the metabolic syndrome group (M) in comparison to controls (Figure 1C). Administering either AM6545 (M + A65) or AM4113 (M + A41) to the metabolic rats had no effect on the creatinine clearance (Figure 1C).

### 2.3. Effect of AM6545 and AM4113 Treatments on Blood Pressure

Measurement of blood pressure using the non-invasive tail cuff method, revealed that MetS animals (M, M + A65, M + A41) showed significantly higher systolic and diastolic blood pressures in comparison to control animals. Treatment of the animals with either AM6545 or AM4113 did not cause any significant changes in the systolic and diastolic blood pressures (Table 2).

### 2.4. Impact of AM6545 and AM4113 Treatments on Renal Histopathology in MetS Rats

**Figure 2:**
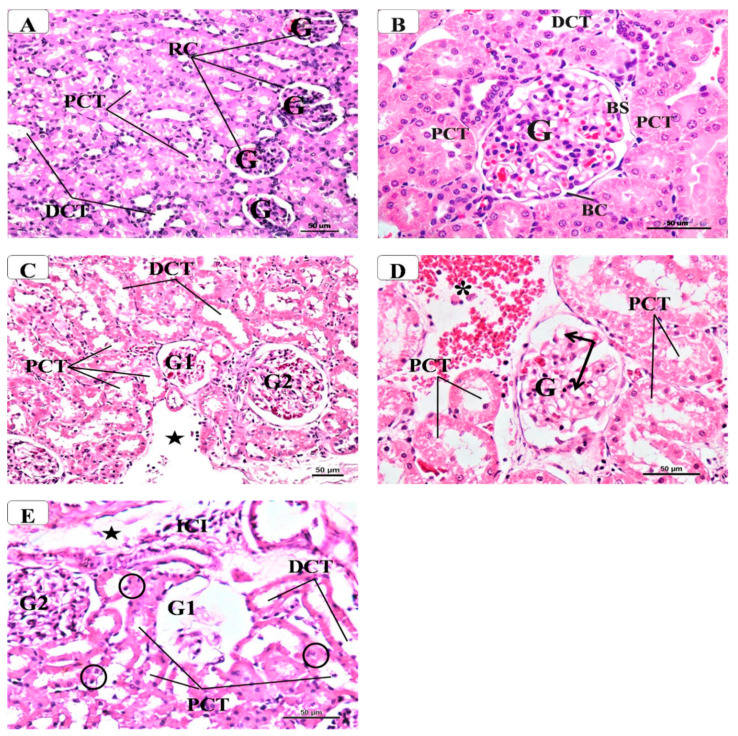
Representative photomicrographs of the histological structure of renal cortex in control and metabolic syndrome rats. Control group (**A**,**B**) shows normal renal glomeruli (G) and tubules (PCT, DCT), while in the metabolic syndrome group (**C**–**E**) some glomeruli appeared atrophied (G1) while others appeared hypertrophied (G2) with dilated capillaries (↑). Most of the PCT appeared degenerated with widened lumen, loss of brush border and pyknotic nuclei (inside circle). Note the presence of interstitial wide spaces (⋆), blood extravasation (*) and infiltration with inflammatory cells (ICI). (H&E—**A**,**C** × 200—**B**,**D**,**E** × 400).

**Figure 3:**
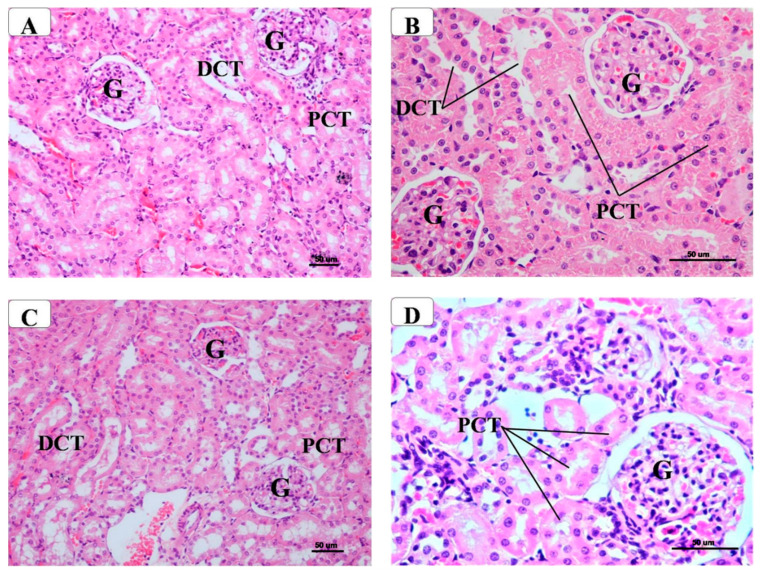
Representative photomicrographs of the histological structure of the renal cortex in the rat’s groups of metabolic syndrome treated with AM6545 (**A**,**B**) and AM4113 (**C**,**D**) CB1 receptor antagonists. Notice the restoration of nearly normal appearance as compared to the control group regarding the glomeruli (G) and tubules (PCT, DCT). (H&E—**A**,**C** × 200—**B**,**D** × 400).

Examination of H&E-stained sections from MetS rats showed marked histopathological changes, especially in the renal cortex where abnormal changes were seen mainly in the glomeruli and proximal convoluted tubules and renal stroma relative to the control group. The kidney of control rats displayed the normal structure of the renal cortex (Figure 2A,B), which contained mostly renal corpuscles consisting of glomerular capillaries enclosed by Bowman’s capsule. Further, there was a capsular space between the proximal (PCT) and distal convoluted tubules (DCT). Tall cuboidal cells having an eosinophilic cytoplasm with central rounded nuclei lined the proximal convoluted tubules. Tall microvilli filled the lumen of the tubules, which showed a brush luminal border. The distal convoluted tubules were scarce relative to the PCT. In MetS rats, the kidney showed variable degrees of changes (Figure 2C–E) where some glomeruli were hypertrophied with dilated glomerular capillaries while other glomeruli appeared atrophic. Additionally, most PCTs were dilated with loss of brush borders; their cells had vacuolated cytoplasm and dark pyknotic nuclei. Moreover, the DCT displayed some degenerative changes with walls and vacuolated cytoplasm. In addition, interstitial spaces, peritubular hemorrhage and mononuclear inflammatory cellular infiltration were seen in different areas of the renal cortex. Interestingly, MetS rats treated with AM6545 (Figure 3A,B) and AM4113 (Figure 3C,D) showed a marked improvement in the renal histological profile manifested by nearly normal glomeruli and PCT without signs of degeneration and/or inflammatory cells infiltration.

### 2.5. Effect of AM6545 and AM4113 Treatments on Uric Acid Levels in MetS Rats

**Figure 4:**
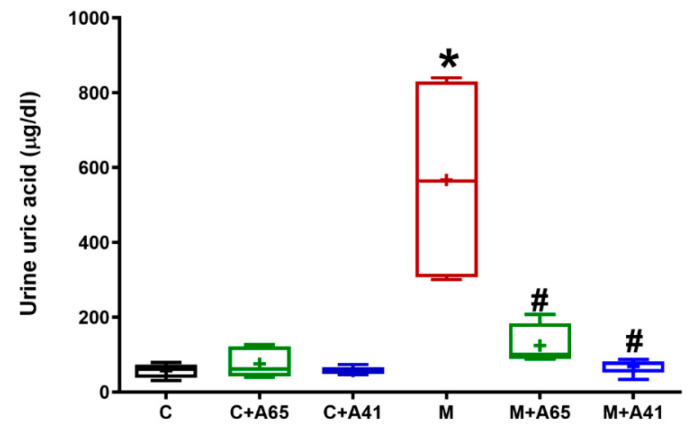
The CB1 receptor antagonists AM6545 and AM4113 blocked the tenfold elevation in urine uric acid content in high-fructose high-salt induced metabolic syndrome rats. The concentration of uric acid was measured in urine of the control (C) and metabolic syndrome (M) rats as well as control and metabolic rats treated with AM6545 (C + A65) and (M + A65) respectively or AM4113 (C + A41) and (M + A41) respectively at a dose of 10 mg/kg/day. Results are shown as box plots and the mean is shown as (+) (*n* = 8). * *p* < 0.05 relative to the control group and # *p* < 0.05 relative to the metabolic syndrome group by one-way ANOVA followed by Tukey’s post hoc test.

Given the importance of uric acid in kidney inflammation and progression to chronic kidney disorder, Figure 4 shows that MetS animals had a ten-fold increase in the urine uric acid content relative to controls (*p* < 0.05). However, both CB1 antagonists, AM6545 and AM4113, significantly (*p* < 0.05) inhibited this increased uric acid content while restoring it to near normal values. Meanwhile, neither AM6545 nor AM4113 significantly affected the urine content of uric acid in control animals.

### 2.6. Effect of AM6545 and AM4113 Treatments on TGFβ1 Levels in MetS Rats

**Figure 5:**
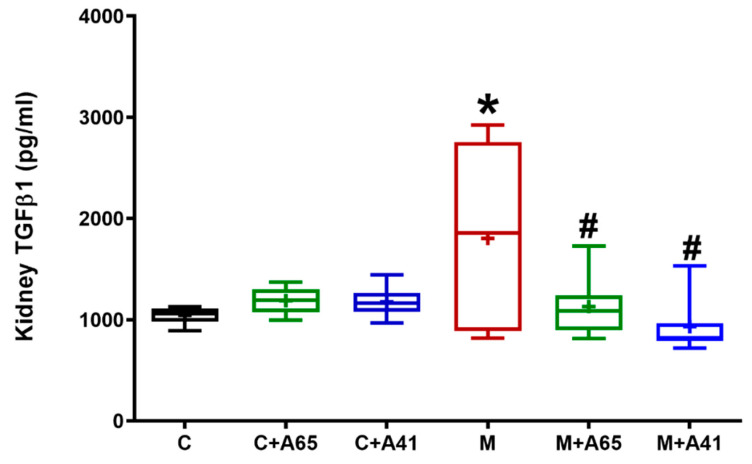
The CB1 receptor antagonists AM6545 and AM4113 reduce TGFβ1 production in fructose-induced metabolic syndrome rats. The concentration of TGFβ1 was measured in the control (C) and metabolic syndrome (M) rats as well as control and metabolic rats treated with AM6545 (C + A65) and (M + A65), respectively, or AM4113 (C + A41) and (M + A41), respectively, at a dose of 10 mg/kg/day. Results are shown as box plots and the mean is shown as (+) (*n* = 8). * *p* < 0.05 relative to the control group and # *p* < 0.05 relative to the metabolic syndrome group by one-way ANOVA followed by Tukey’s post hoc test.

The study further investigated the role of TGFβ1 as a major mediator of pro-inflammation and fibrosis. As shown in Figure 5, tissue concentration of TGFβ1 in kidneys isolated from MetS rats were markedly (*p* < 0.05) increased than in normal animals. However, the four weeks of treatment with AM6545 and AM4113 resulted in a significant (*p* < 0.05) reduction of TGFβ1 concentrations, restoring it to normal values. While neither AM6545 nor AM4113 significantly affected TGFβ1 tissue concentrations in control kidneys. This result suggests that the CB1 receptor has a critical role in mediating inflammation and kidney fibrosis.

The correlation coefficient was computed to evaluate the correlation between the albumin excretion rate and the kidney level of TGFβ1 (Table 3). A strong correlation was present between the AER and TGFβ1 in control and metabolic syndrome animals. The correlation was also present in the animals treated with AM6545, but not with AM4113.

### 2.7. Effect of AM6545 and AM4113 Treatments on Renal Fibrosis in MetS Rats

**Figure 6:**
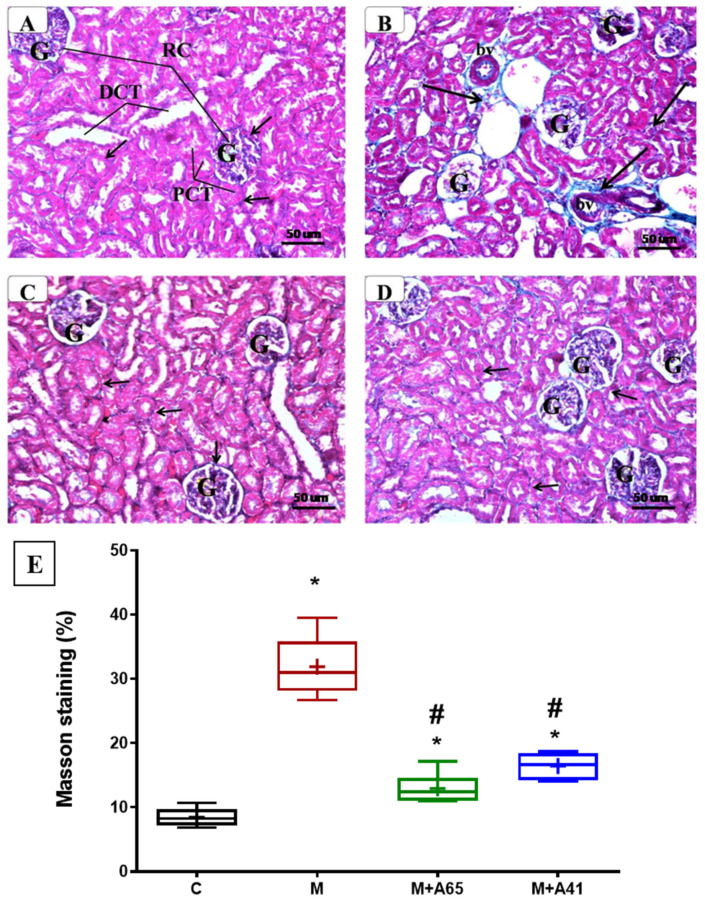
Representative photomicrographs of renal cortex of different groups stained with Masson’s Trichrome. Notice the marked increase of collagenous fibers (↑) in the kidney of metabolic syndrome (**B**) around the glomeruli (G) and tubules (PCT, DCT) as compared to the control (**A**). In contrast, AM6545-treated metabolic syndrome (**C**) and AM4113-treated metabolic syndrome (**D**) showed a noticeable reduction of collagenous fibers. (Masson’s Trichrome, A, B, C & D × 200). (**E**) shows the quantification of Masson’s Trichrome staining expressed as a %. Results are shown as box plots and the mean is shown as (+) (*n* = 8). * Significantly different from “C” at *p* < 0.05, # Significantly different from “M” at *p* < 0.05.

Figure 6 shows the kidney sections from different groups stained by Masson’s Trichrome to assess the collagenous fibers, which appeared as a bluish coloration. The renal cortex showed a marked increase of collagenous fibers in kidneys taken from MetS rats (Figure 6B) as compared to the control rats (Figure 6A). In the interstitium and glomeruli, there was a surplus of collagen fiber deposition. In contrast, there was reduced deposition of collagenous fibers in the renal cortex with AM6545 (Figure 6C) and AM4113 (Figure 6D) treatments. Quantification of Masson’s Trichrome staining revealed a higher percentage in the metabolic syndrome (M) as well as in the metabolic treated groups (M + A65, M + A41) in comparison to controls. However, treatment of MetS animals with AM6545 and AM4113 caused a significant lowering of Masson’s staining relative to the non-treated metabolic group.

## 3. Discussion

The development of nephropathy is a serious complication of MetS that is currently increasing worldwide. AM6545 and AM4113 are both neutral antagonists for the CB1 receptor, which has been shown to be hyper-activated in MetS [27]. In this study, we have examined the effect of these drugs on MetS-induced nephropathy. The increased risk of developing kidney disease in MetS has been well documented [6,7,8]. We have recently demonstrated that eight weeks of high-fructose and high-salt loading in Wistar rats was sufficient to induce a state of MetS manifested by obesity, hyperinsulinemia, hyperuricemia and dyslipidemia [28]. Furthermore, we have demonstrated that pre-treatment of these animals with either AM6545 or AM4113 caused an alleviation of the developed metabolic syndrome. This was demonstrated by an inhibition of insulin resistance, decreased body weight, anti-dylipidemic, anti-hyperurecemic as well as anti-inflammatory actions. We have demonstrated that the area under the curve of the oral glucose tolerance test following an oral challenge of glucose was higher in the metabolic syndrome group relative to the controls. Effects on blood glucose levels were ameliorated after either AM6545 or AM4113 administration [28]. Moreover, studies by members of our group have shown that both AM6545 and AM4113 reduce food intake in rodents and thus can be useful in the treatment of obesity [22,29].

The high-fructose high-salt model of MetS was shown to induce morphological changes in the kidneys and marked elevation in renal injury markers [10]. In the current study, we have found that MetS rats had an increased endocannabinoid tone as shown by the elevated anandamide and 2-AG amounts in the kidney. Therefore, we hypothesized that using the CB1 antagonists AM6545 and AM4113 could relieve the enhanced tone and ameliorate kidney functional parameters. Although metabolic syndrome rats had significantly higher blood pressures compared with control rats, the administration of neither AM6545 nor AM4113 had no further effect on blood pressure in the present model. This is consistent with a recently published study by members of our group, which reported that AM6545 did not cause any change in blood pressure when administered to streptozotocin-induced diabetic mice [30]. Some studies have reported that blocking CB1 receptors systemically improved blood pressure in hypertensive, insulin resistant and obese rats [31,32]. However, the effects of AM6545 and AM4113 on blood pressure and the cardiovascular system is an area requiring further investigation. The findings of our study and of Barutta et al. (2018) suggest that AM6545 and AM4113 may be producing their effects on the kidney independently of blood pressure [18].

In the present study, assessment of kidney morphology revealed that untreated MetS rats displayed glomerular, tubular and stromal injuries in the renal cortex. In accordance, Alderson et al. (2004) attributed the enlarged glomeruli which were found in our study to structural and functional adaptations. This eventually leads to hyperfiltration in the nephrons, and is commonly reported in focal segmental glomerulosclerosis [33]. Additionally, there were several glomeruli which displayed atrophy and an enlarged capsular space. This is in accordance with other studies, which have classified this glomerulus as shrunken and sclerotic [34].

This study also looked at the effect on the PCT, particularly the tubular cells. This is where the fructose transporters (GLUT-2 and GLUT-5) are found as well as the location in which the enzyme ketokinase is expressed. Ketokinase has been reported as a major enzyme in fructose metabolism [35]. The dilation of the tubles and cytoplasmic vacuolation were consistent with previous findings, which found that chronic tubulointerstitial nephropathy leads to the formation of cysts and dilatation of the cortex tubules [34]. Perhaps the infiltration of cells reported in this study represents a mechanism by which the kidney defends itself and rapidly removes necrotic tissues. In addition, there is a halt in the circulation due to congestion, which ultimately increases the blood capillaries’ permeability causing red blood cell extravasation. Furthermore, fructose is known to cause inflammation in the rat renal tissues as well as to stimulate inflammation in the endothelium [36].

In the current study, there was excess presence of collagen fibers in the kidneys of MetS rats. This is in agreement with other findings which suggest that collagen fiber accumulation is presents in patients having proteinurea. They also reported that this is classically seen in patients with glomeruloscelerosis and could lead to glomerular degeneration. Furthermore, it was reported that very little glomerular capillaries were found in sclerotic lesions, with collagen deposition and accumulation of mesangial matrix [37].

CB1 receptor activation was previously shown to affect renal fibrogenesis [38]. Consistent with this notion, MetS rats in the current study exhibited tissue fibrosis in the renal stroma relative to control rats (Figure 4). This was further explained by the significant elevation in TGFβ1 levels (Figure 5). TGFβ1 is an inflammatory and profibrogenic cytokine, which is involved in the pathological process of kidney fibrosis [39,40]. Importantly, treatment of MetS rats with AM6545 and AM4113 significantly reduced renal TGFβ1 levels, indicating that CB1 could be a possible downstream mediator of TGFβ1 signaling. Collectively, the reduction in TGFβ1 is tied with reduced inflammatory responses reversing the fibrotic responses and morphological renal changes discussed above. A correlation was found between AER and levels of TGFβ1 in the kidney in untreated animals and in animals treated with AM6545 but not AM4113. This suggests that AM4113 could be acting slightly differently than AM6545.

Another significant finding is that treatment of MetS rats with AM6545 and AM4113 significantly improved renal function via the reduction in renal fibrosis. This included a significant reduction in urinary uric acid excretion, albumin excretion rate and urinary protein excretion. These findings are in accordance with previous studies, indicating that CB1 blockade with rimonabant had significantly improved renal functional parameters in obese *fa*/*fa* Zucker rats [14]. Briefly, Janiak et al. (2007) showed that long term rimonabant administration significantly improves creatinine clearance, slows proteinuria development and lowers glomerular and tubular lesions severity. In the present study, we report that AM6545 and AM4113 inhibited the elevated proteinuria and albumin excretion rate while having no effect on creatinine clearance.

Alternatively, the therapeutic effects of AM6545 and AM4113 antagonists in renal function might also result from the correction of the underlined MetS disorder. Several reports have demonstrated that dyslipidemia, including high triglycerides and high serum cholesterol levels, may contribute to kidney disease [41,42]. In our previous study, CB1 blockade with both antagonists produced a marked decrease in serum cholesterol and triglycerides, serum insulin, body weight, serum uric acid and liver TNFα together with increased adiponectin levels in the same MetS rat model [28]. Therefore, these beneficial effects could indirectly be preserving renal function. In the future, it would be interesting to examine whether these CB1 antagonists could be therapeutically useful in kidney disease unrelated to MetS.

## 4. Materials and Methods

### 4.1. Chemicals

Synthesis of AM6545 5-(4-[4-cyanobut-1-ynyl]phenyl)-1-(2,4-dichlorophenyl)-4-methyl-*N*-(1,1-dioxo-thiomorpholino)-1*H*-pyrazole-3-carboxamide and AM4113 (5-(4-alkylphenyl)-1-(2,4-dichlorophenyl)-4-methyl-*N*-(piperidin-1-yl)-1*H*-pyrazole-3-carboxamide) was carried out in our lab at the Center of Drug Discovery, Northeastern University, Boston, MA, USA, as previously outlined [22,29].

### 4.2. Animals

A number of six-week-old male Wistar rats (150–190 g) were obtained from King Abdulaziz University (KSA) and were held under a 12-h light: 12-h dark cycle at room temperature (20 ± 2 °C). The animals were given free access to water and were fed with standard rat chow ad libitum for 7 days of acclimatization prior to the experiment. Experimental procedures were conducted according to the Guidelines for Animal Care and Treatment of the Biomedical Ethics Research Committee at King Abdulaziz University (Reference No. 479–16). The same animals used in our previous published work were used in the current study [28].

### 4.3. MetS Model and Experimental Design

Rats were placed randomly into six groups (*n* = 8/group), as previously described in our published work [28]. Briefly, the first control group (C) was fed a standard rodent food with freely accessible water for eight weeks. The second and third groups were control groups treated with AM6545 (C + A65) or AM4113 (C + A41) drugs. The fourth group, MetS group (M), was fed a standard rodent food but had free access to 20% *w/v* fructose solution + 3% salt throughout the same period. The fifth and sixth groups were MetS groups treated with AM6545 (M + A65) or AM4113 (M + A41) drugs. Both drugs were administered as a suspension in 0.5% carboxymethyl cellulose (CMC) by intraperitoneal (IP) injection at a dose of 10 mg/kg/day for another four weeks. Control and MetS groups were only given CMC intraperitoneally as vehicle control. Development of MetS was proven by the insulin resistance, obesity, elevated adiponectin level and dyslipidemia as shown in our published work [28].

### 4.4. Specimen Collection

At the end of 12 weeks, each rat was placed in a single metabolic cage for 1 day for collection of the urine while having free access to food and water. Uric acid, albumin excretion rate (AER) and total protein concentrations were determined from urine samples. After that, IP urethane (1 g/kg) was used to anesthetize the rats. Kidneys were rapidly removed and immediately stored in −80 °C for biochemical measurements and histological examination.

### 4.5. Tissue Homogenates

Renal specimens were minced and manually homogenized using an electrically driven ceramic pestle in homogenization buffer (phosphate buffer saline + 1% Triton ×100, pH 7.4). The homogenates were centrifuged at 4000× *g* at 4 °C for 20 min and the clear supernatant was frozen and held at −80 °C until further processing.

### 4.6. Measurement of Biochemical Parameters

The concentration of transforming growth factor β1 (TGFβ1) was determined in kidney tissue homogenates by enzyme-linked immunosorbent assay (ELISA) using Rat TGF beta 1 Kit (ab119558). An optical density using an automated ELISA reader was used for quantification. The urinary uric acid, albumin excretion rate and proteinuria levels were measured calorimetrically using Crescent kits (Crescent diagnostic, Jeddah, KSA), according to the protocols specified by the manufacturer. The endocannabinoids AEA and 2-AG were measured in kidney homogenates by kits from Real-Gene Labs (Lakeforest, CA, USA).

### 4.7. Measurement of Blood Pressure

Blood pressure was measured non-invasively by the tail-cuff procedure. Animals were slightly restrained while conscious and kept warm with daily conditioning for 15 min for 3 days. Blood pressure was recorded in the morning between 8 and 10 a.m. The procedure consisted of 10 automatic inflation/deflation cycles which were repeated 10 times with a 5–10 min stabilization period in a 35 °C chamber. Blood pressure was taken as the average of 6 recordings in the range of 5–10 mmHg.

### 4.8. Calculation of Creatinine Clearance

Creatinine clearance was calculated as [43]:

Urine creatinine/Serum creatinine × (Urine Volume (mL)/Time (h) × 60)

### 4.9. Assessment of Kidney Histopathology

An amount of 10% formalin was used to fix kidney samples, which were processed and embedded in paraffin wax. Paraffin sections of 5 µm thick were fixed onto glass slides and stained with hematoxylin and eosin (H&E) in accordance with standardized protocols to examine the renal tissue histology and Masson’s trichrome to assess collagen fibers content. Kidney sections were examined and photographed using light microscope (Nikon Eclipse TE2000-U, NIKON, Tokyo, Japan). For determination of % staining with Masson trichrome, six sections from the kidney *n* each group were photographed and subjected to analysis using Image J analysis software (ImageJ, 1.46a, National Institutes of Health, Bethesda, Maryland, USA) as follows:

$$\%\;{\mathrm{Masson}}^{\prime }s\;\mathrm{trichrome}\;\mathrm{staining}\;=\;\frac{\mathrm{Area}\;\mathrm{stained}\;\mathrm{with}\;{\mathrm{Masson}}^{\prime }s\;\mathrm{trichrome}\;}{\mathrm{Total}\;\mathrm{area}\;\mathrm{assessed}}\times 100$$

### 4.10. Data and Statistical Analysis

Data are shown as the mean ± SE of mean and the data distribution is given as box plots. Results from different groups were analyzed by “Kolmogorov–Smirnov” normality test one-way analysis of variance (ANOVA) followed by Tukey’s post hoc test using GraphPad Prism (Version 8). *p* < 0.05 was considered statistically significant. Analysis of correlation (parametric) was used to calculate the correlation coefficient and *p* value between AER and kidney level of TGFβ1 using the same software.

## 5. Conclusions

In summary, the CB1 receptor neutral antagonists, AM6545 and AM4113, inhibited the raised proteinuria and albumin excretion rate without affecting creatinine clearance or blood pressure. We have shown that blockade the CB1 receptor with both drugs reversed the histopathological changes induced by high-fructose high-salt feeding in renal tissue. Furthermore, both drugs produced a significant decrease in the renal pro-inflammatory cytokine TGFβ1. Collectively, these results indicate that AM6545 and AM4113 have reno-protective effects in this MetS animal model, raising hope for their future potential therapeutic intervention in a variety of kidney diseases.

## Figures and Tables

**Table 1:** Kidney content of anandamide and 2-arachidonoylglycerol.

	Control (C)	C + A65	C + A41	MetS (M)	M + A65	M + A41
Anandamide (pmol/g)	1.15 ± 0.12	1.32 ± 0.17	1.38 ± 0.19	2.31 * ± 0.28	2.17 * ± 0.24	2.33 * ± 0.20
2-Arachidonoylglycerol (nmol/g)	3.57 ± 0.38	3.33 ± 0.35	2.98 ± 0.31	5.1 * ± 0.62	4.80 * ± 0.52	4.77 * ± 0.55

Data are presented as Mean ± SD (*n* = 8). Statistical analysis was performed by two-way ANOVA followed by Tukey’s test. * Significant difference from control group at *p* < 0.05.

**Table 2:** Effect of AM6545 and AM4113 on systolic and diastolic blood pressure (BP) of metabolic syndrome (MetS)-induced rats.

	Control (C)	C + A65	C + A41	MetS (M)	M + A65	M + A41
Systolic BP (mmHg)	112 ± 7.50	111 ± 12.2	114 ± 11.8	138 * ± 12.1	130 * ± 7.8	128 * ± 8.1
Diastolic BP (mmHg)	71 ± 8.1	73 ± 6.9	75 ± 8.2	92 * ± 8.7	89 * ± 6.4	84 * ± 5.4

Data are presented as Mean ± SD (*n* = 8). Statistical analysis was performed by two-way ANOVA followed by Tukey test. * Significant difference from control group at *p* < 0.05.

**Table 3:** The effects of AM6545 and AM113 on correlation between albumin excretion rate (AER) and kidney level of the transforming growth factor beta 1 (TGFβ1).

	Correlation Coefficient (r)	*p*-Value
Untreated animals (C, M)	0.596	0.041 *
A65 treated animals (C + A65, M + A65)	0.712	0.048 *
A41 treated animals (C + A41, M + A41)	0.230	0.552

* Statistically significant correlation at *p*< 0.05.

## Data Availability

Data is contained within the article.

## Sample Availability

Samples of the compounds are available from the authors.
