# Correction: Eid et al. Interference with TGFβ1-Mediated Inflammation and Fibrosis Underlies Reno-Protective Effects of the CB1 Receptor Neutral Antagonists AM6545 and AM4113 in a Rat Model of Metabolic Syndrome. *Molecules* 2021, *26*, 866

**DOI:** 10.3390/molecules29040902

**Published:** 2024-02-19

**Authors:** Basma G. Eid, Thikryat Neamatallah, Abeer Hanafy, Hany M. El-Bassossy, Lenah Binmahfouz, Hibah M. Aldawsari, Atif Hasan, Gamal Abd El-Aziz, Kiran Vemuri, Alexandros Makriyannis

**Affiliations:** 1 Department of Pharmacology and Toxicology, Faculty of Pharmacy, King Abdulaziz University, Jeddah 21589, Saudi Arabia; taneamatallah@kau.edu.sa (T.N.); ahanafyvet@yahoo.com (A.H.); lbinmahfouz@kau.edu.sa (L.B.); 2 Department of Pharmacology, Faculty of Veterinary Medicine, Kafrelsheikh University, Kafrelsheikh 33516, Egypt; 3 Department of Pharmacology and Toxicology, Faculty of Pharmacy, Zagazig University, Zagazig 44519, Egypt; helbassossy@pharmacy.zu.edu.eg; 4 Department of Pharmaceutics, Faculty of Pharmacy, King Abdulaziz University, Jeddah 21589, Saudi Arabia; haldosari@kau.edu.sa; 5 Department of Anatomy and Embryology, Faculty of Veterinary Medicine, Kafrelsheikh University, Kafrelsheikh 33516, Egypt; atifhasan178@gmail.com; 6 Department of Anatomy, Faculty of Medicine, King Abdulaziz University, Jeddah 21589, Saudi Arabia; dr_gamal_said@yahoo.com; 7 Center for Drug Discovery, Northeastern University, Boston, MA 02115, USA; kiranvvemuri@gmail.com (K.V.); a.makriyannis@northeastern.edu (A.M.); 8 Departments of Chemistry and Chemical Biology and Pharmaceutical Sciences, Northeastern University, Boston, MA 02115, USA

The authors wish to make the following changes to the paper [1]:

In the original publication, there was a mistake in Figure 6 as published. Human error may have occurred during assembly of the subfigures; therefore another representative picture has been chosen for panel D. The corrected Figure 6 appears below. The authors apologize for any inconvenience caused and state that the scientific conclusions are unaffected. The original publication has also been updated.

## Figures and Tables

**Figure 6:**
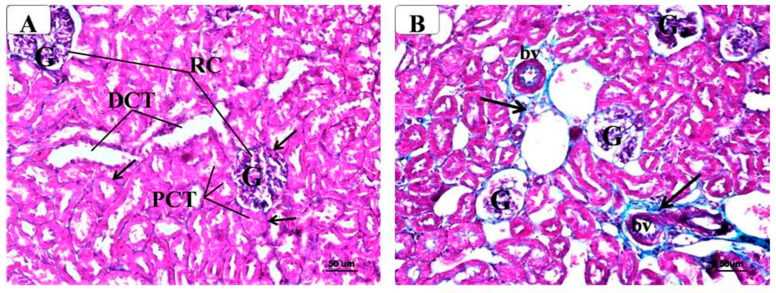

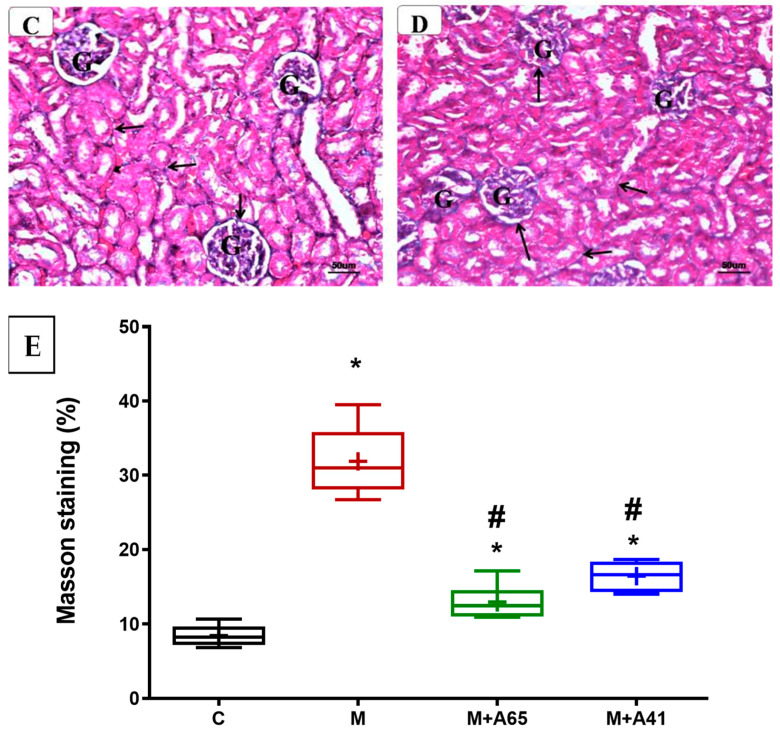
Representative photomicrographs of the renal cortex of different groups stained with Masson’s Trichrome. Note the marked increase in collagenous fibers (↑) in the kidney of metabolic syndrome (**B**) around the glomeruli (G) and tubules (PCT, DCT) as compared with the control (**A**). In contrast, AM6545-treated metabolic syndrome (**C**) and AM4113-treated metabolic syndrome (**D**) showed noticeable reductions in collagenous fibers. (Masson’s Trichrome, A, B, C, and D × 200). (**E**) The quantification of Masson’s Trichrome staining expressed as a percentage. The results are shown as box plots; the means are shown as (+) (*n* = 8). * Significantly different from “C” at *p* < 0.05, # Significantly different from “M” at *p* < 0.05.
